# Tourism Destination Management Strategy for Young Children: Willingness to Pay for Child-Friendly Tourism Facilities and Services at a Heritage Site

**DOI:** 10.3390/ijerph17197100

**Published:** 2020-09-28

**Authors:** Hwasung Song, Chanyul Park, Miseong Kim

**Affiliations:** 1 Department of Urban management, Suwon Research Institute, Suwon 16829, Korea; hssong@suwon.re.kr; 2 Public and Private Infrastructure Investment Management Center, Gyeongnam Institute, Changwon 51430, Korea; chan10a@gmail.com; 3 Smart Tourism Education Platform, KyungHee University, Seoul 02447, Korea

**Keywords:** tourism destination management, young children’s tourism, accessible tourism, facilities and services, choice experiment, UNESCO World Heritage Site, health/hygiene and amenities

## Abstract

The present study seeks to establish tourism destination management centered on young children for accessible tourism from a public perspective. Preferences for services and facilities for young children were identified using a choice experiment (CE). The present study was conducted at Hwaseong Fortress, a UNESCO World Heritage Site, located in Suwon City, which leads the clean restroom culture. Overall, 1870 experiments were conducted with 374 participants to estimate their willingness to pay for child-friendly tourism facilities and services. As a result, willingness to pay was found in the order of specialized courses for young children, rest areas, restrooms, and rides. In conclusion, the importance of health/hygiene and amenities has implications for tourism destination management for young children. This study contributes to a better understanding of families with young children by evaluating preferences for levels of services and facilities for young children.

## 1. Introduction

Have you ever experienced tourism with young children? In the Republic of Korea, under Article 2 of the Child Care Act, infants and young children refers to preschoolers under the age of six [1]. Tourism with a child under 6 years of age brings many restrictions at tourist destinations. During tourism with young children, one faces the constraints of the basic needs for rest, eating, and toileting [2,3,4]. Thus, it is expected that the contents that can be enjoyed at tourist destinations will change.

The impact of children on tourism purchasing power is gradually increasing, and family vacations are expected to become more important for society in the future from the perspective of creating social benefits [5]. Parents are gradually spending more money and energy to travel with their children [6]. Additionally, travel experiences during one’s childhood could affect travel behavior after becoming an adult [7,8,9]. Therefore, not only is the scale of young children’s tourism growing, but its importance in marketing is also increasing [9]. 

From these perspectives, research on travel time and cost for travel with young children [6], preferred activities [4,10,11], and facilities and services [2,3,12,13,14] has been steadily progressing. These studies have contributed to a better understanding of the tourism market for young children, but there is still a lack of answers as to which management strategy is needed from the view of providers. In particular, in terms of safety and hygiene, it is necessary to consider how to differentiate food, sleeping, facilities and services, and experiences. This is a more difficult aspect to deal with in the public domain. However, since tourism is a right that everyone should enjoy, not only an understanding of the economic importance of young children’s tourism, but also the development of a tourism destination management strategy for young children’s tourism in the public domain is needed. 

Accessible tourism for all means “the ongoing endeavor to ensure tourist destinations, products, and services are accessible to all people, regardless of their physical limitations, disabilities, or age” [15]. Young children, who have more restrictions than adults in terms of hygiene, safety, walking, and rest, are also the subject for accessible tourism. That is, the public sector should also improve facilities and services for young children. 

The tourism industry has been negatively impacted due to the COVID-19 pandemic, and long-distance tourism such as overseas tourism has become very difficult [16,17]. However, tourism and leisure are basic needs of humans [18], and as COVID-19 has become prolonged and new cases have significantly decreased, travel to nearby destinations such as parks is rapidly recovering in Korea [19]. However, COVID-19 makes tourism activities difficult for young children with weak immune systems. Tourists with young children are very concerned about the fatigue of their children, so the younger the children are, the more they prefer short distances and try to reduce travel time as much as possible [4,10]. In addition, the comfort and safety of the destination is an important determinant of the intention to visit, and the cleanliness of the destination is especially important [12]. This is the reason why COVID-19 further reduces tourism activities, especially for tourists with young children. In the Republic of Korea, childcare facilities have been closed due to COVID-19, and children spend most of their time at home. Social exchanges and education provided by childcare facilities are on hold, tourism and outdoor activities are reduced, and people are suffering from increases in obesity due to mental stress and lack of exercise [20]. In particular, children belonging to the socially vulnerable class are more alienated, mentally stressed, and worsening in their physical health [21]. Accordingly, it is necessary to look at young children’s tourism from the perspective of accessible tourism in terms of public interest and to improve the conditions of young children’s tourism to help heal the society after the COVID-19 pandemic. Under these circumstances, the importance of health/sanitation management for young children’s tourism is growing [16].

Therefore, the present study examines the preferences of tourists in order to establish a tourism destination management strategy for young children as a type of accessible tourism from a public perspective. Choice experiments (CEs) were used to estimate the willingness to pay (WTP) for facilities and services for young children at a tourist destination, in order to understand preferences. The CE method of estimating economic value based on stated preferences is widely used for estimating the WTP of tourists. The advantage of the CE is that you can check the WTP for the different levels of any attribute. For example, it is possible to check how much the WTP is based on the level of restrooms for young children. 

Suwon Hwaseong Fortress, the target site of this study, was designated as a UNESCO Cultural Heritage Site in 1997 and is a popular historical and cultural tourist destination in Korea, even among young children, due to its educational aspects [22]. In particular, Suwon City, in which Hwaseong Fortress is located, is the place where the “World Restroom Culture Movement” began and is well known for its clean public restrooms [23]. Yet, facilities and services for young children including restrooms at the tourist destination are insufficient. Thus, this site was selected as the target site for establishing an optimal tourism destination management strategy for young children’s tourism. 

The purpose of the present study was to establish a tourism destination management strategy centered on facilities and services for young children’s tourism before the outbreak of COVID-19. However, hygiene and amenities in young children’s tourism and providing preferred facilities and services has become even more relevant in the era of COVID-19, with implications for the tourism destination management for young children’s tourism.

## 2. Tourism Destination Management for Young Children

### 2.1. Importance of Young Children in the Tourism Industry and Their Characteristics as Tourists

Young children are an important consumer group in the tourism industry. Tourism has become one of the essential consumption elements for families with young children, and parents are spending more and more money and energy on travel with their children [6,8]. Children have a fair amount of influence on the tourism decision-making in the family. Therkelsen revealed that the roles of mothers and children are equal in vacation planning [4], and Curtale found that parents are sensitive to their children’s best and worst choices, and they make choices tailored to their preferences [9]. In addition, the satisfaction of children affects the satisfaction and behavior intention of parents [7,9]. In particular, younger children have a greater influence on tourism decision-making [9,10].

The size of the domestic children’s industry in Korea was 40 trillion in 2018, surpassing the 27 trillion from 2012, and new words such as VIB (Very Important Baby) and Yucance (Infant + hotel + vacation) were born [24]. In the tourism sector, in particular, various specialized programs are being provided for these VIB customers. For example, worldwide resort Club Med provides various activities and facilities that are adapted to children of different age groups, ranging from 4 months to 17 years [25]. Even in Korea, Midas Hotel and Resort [26] was designed for vacationing with children and operates premium children’s clubs in line with this trend. Therefore, in the tourism industry, it is necessary to establish marketing strategies for children in order to attract new customers, and it is important to provide tourism products that satisfy the preferences of children [9].

The private sector focuses on the market value of the children’s industry, while the public sector focuses more on creating child-friendly tourism environments from an accessible tourism perspective [24]. Since tourism is increasingly being conceived as a necessity, rather than a luxury [27], accessible tourism is being considered both as a fundamental right as well as a new business opportunity [28]. Accessible tourism for all means “the ongoing endeavor to ensure tourist destinations, products, and services are accessible to all people, regardless of their physical limitations, disabilities, or age” [15]. It could also be understood as the set of “facilities and services (including the physical environment, transportation, information, communication) which enable persons with special access needs, either permanent or temporary, to enjoy a holiday and leisure time with no particular barrier or problem” [29]. From this perspective, it has become important to consider the level of accessibility for young children when designing facilities and services of tourist destinations.

In order to create child-friendly destinations, it is necessary to understand the characteristics of young children as tourists. First, young children need to have their primary needs for food, nap/bedtime, safety, and sanitary facilities met first [4]. Families with young children will give positive reviews on tourism attraction if they are provided convenience facilities and food for their children [3]. Nistoreanu and Dragolea found that specialized facilities for children such as beds for children or rest areas are needed for providing comfort, and caring staff and hygienic conditions could improve safety [12]. In addition, Khoo-Lattimore and Yang emphasized that a comprehensive approach should be taken when designing facilities and services for young children by considering accessibility, possibility of interaction with other children, safety, hygiene, room size, and staff size for child-friendly programs [14]. Secondly, young children are usually satisfied with dynamic activities. Nickerson and Jurowski revealed that young children prefer dynamic activities such as gold mining more than static activities such as seeing an exhibition [11]. Young children need engaging activities including various ecological experiences such as picking fruit, playing with animals, and fishing [12]. In addition, children prefer dramatic play opportunities such as horse riding and superheroes, and functional play opportunities such as swinging and rocking [30]. Cosco found that children enjoy riding on wheeled toys on hard, curvy pathways [31], and Babara found that most observed play focused on standing, walking, running, and riding [32]. In sum, children need facilities that are comfortable and safe, and dynamic activities that are fun and entertaining.

### 2.2. Specialization of Facilities and Services for Young Children at Tourist Destinations

Due to the characteristics of young children as previously discussed, facilities and services at tourist destinations must also be designed for young children. For families with young children, tourist attractions should provide customized facilities and services for children to minimize problems and to maximize their satisfaction during the tour [12,13]. When comfort and safety are secured, families with young children are willing to visit tourist destinations. Parents consider a safe and clean environment as the most important factor for choosing destinations while accompanied by young children [3,13]. Asians, in particular, regard clean restrooms as more important than Hispanic or African Americans [33]. Furthermore, the worldwide pandemic situation due to COVID-19 has highlighted cleanliness as the most important aspect which could make relieve visitors of anxiety and instead attract them [34]. As handwashing is recognized as an essential activity in maintaining cleanliness, children’s handwashing has increased compared to before the outbreak of COVID-19 [35]. Therefore, it is necessary to prepare facilities (toilets, sinks) that can meet the needs of maintaining cleanliness including hand washing.

Restrooms are also an important facility from the perspective of accessible tourism destinations. Various guidelines for accessible tourism destinations include the criteria of accessibility to public restrooms [15,29,36]. The guidelines of KTO state the importance of the installation of convenience facilities such as nursing rooms and diaper changing stations, and designing these with consideration of children’s characteristics, such as lowering the height of sinks [36]. Regarding the design of restrooms and other convenience facilities, gender parity also needs to be considered. The increase in male participation and co-parenting in early childhood education and care [37], including diaper-changing duties, helps to improve parent-child bonds and family relationships [38]. In this context, male restrooms should also include childcare facilities.

In addition, rest areas such as benches and restroom are important facilities for young children. In order to make children feel safe and comfortable, it is necessary to provide areas for rest and some adequate protection from stressful natural elements such as wind, rain, and sun [12,39]. Even in the view of accessible tourism, having enough accessible rest points such as benches is a basic element [15].

Children’s vehicles should be an additional consideration as well. Various guidelines for accessible tourism destinations regard the secure accessibility of public transportation and accessibility to any tourist spots without difficulties [15,29,36]. For example, a necessary consideration is that young children will usually take strollers into tourist destinations. As Nyman and others revealed that an accessible setting with a universal design could be the reinforcement factor for choosing tourism destinations especially for families with wheelchair-bound children [40], accessibility could be an important factor for travel decision-making for families with young children who should be carried in strollers. In addition, numerous tourist destinations, such as the representative theme park Disney World, provide stroller rental services for families with young children [41]. For providing convenience, stroller or other vehicle rental services should be considered when designing tourist destinations. Providing rides could also add a fun factor to destinations. Various studies have revealed that children love to ride vehicles with wheels, so providing them with vehicles such as baby bicycles, strollers, and minicars could be a specialized option for tourist destinations [30,31,32].

Lastly, specialized programs could strengthen the attractiveness of the destination. As we noted in the previous section, children prefer experiential activities [11,12,30,31,32]. Various tourist destinations provide specialized programs for young children. Numerous museums (e.g., Guggenheim Museum in the United States [42], Remai Modern in Canada [43]) offer stroller tours for families with young children, and some public parks offer playground associate programs for young children (e.g., ‘Kids in Motion’ in New York City parks [44], ‘Playground Program’ in Appleton Parks [45]). These specialized programs may have a positive effect especially during a pandemic era. Specialized programs could broaden the recreation opportunity spectrum (ROS), which is a frequently used management tool for protecting areas [46]. Since it could manage the demand of visitors, it may be used to affect the number of visitors during a particular period in a specific area [47]. In this aspect, specialized programs with limits on time and number of participants, could disperse tourists and lower the density of visitors. This is also a good management plan for reducing the risk of infection by reducing exposure density [48].

## 3. Materials and Methods

### 3.1. Study Area

**Figure 1:**
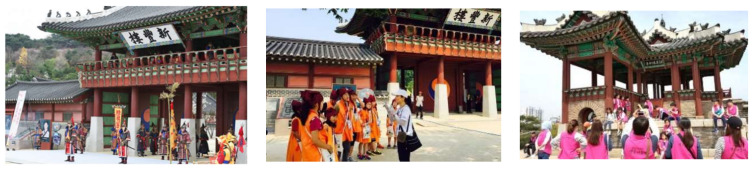
Hwaseong Fortress as a child-friendly place. (Source Indication: [SUWON CITY], [Visit Suwon (http://suwon.go.kr/visitsuwon)]).

Suwon Hwaseong Fortress was recognized for its historical meaning and architectural excellence and designated as a UNESCO Cultural Heritage Site in 1997 [49]. It is the representative historical and cultural legacy of beautiful Asian fortresses, not confined to the Joseon Dynasty in Korea. With various attractiveness, as shown in Figure 1, Hwaseong Fortress is known as one of the child-friendly places. According to the latest tourist survey of Suwon in 2017, the rate of visitors in their 30 s and 40 s at Suwon Hwaseong was 41.7%, much higher than for other age groups. Additionally, the primary type of companions of visitors in their 30 s and 40 s was family members (74.7%), so this group is comprised mainly of families with young children [50]. 

**Figure 2:**
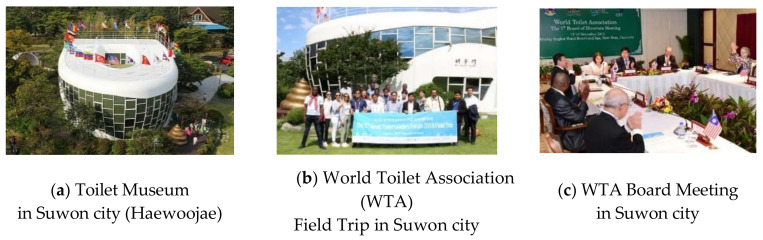
Birthplace of restroom culture, Suwon City. (Source Indication: (**a**) [SUWON CITY], [Visit Suwon (http://suwon.go.kr/visitsuwon)]; (**b**,**c**) [Mr. Toilet House], [Mr. Toilet House, (https://www.haewoojae.com/)].).

Suwon has a large population of young children under the age of 6 [51], and the city is recognized as a city with a child-friendly environment, as it received a child-friendly city (CFC) recognition from UNICEF [52]. In particular, Suwon City, in which Hwaseong Fortress is located, is where the “World Restroom Culture Movement” began and is well known for its clean public restrooms [23]. Suwon is the only city which has a toilet museum (Haewoojae) in Korea and the World Toilet Association is located in Suwon as well. As shown in Figure 2, the city is leading the world restroom culture as the birthplace of restroom culture and plays a pivotal role in the development of restroom culture through the operation of a toilet museum. Therefore, it is the ideal place to evaluate and find the best options for restrooms, the representative convenience facility for tourists, especially in the era of COVID-19 in which hygiene management has become more important. 

### 3.2. Choice Experiment Analysis

In this study, CE analysis was used for investigating preferences for child-friendly tourism facilities and services. The CE is a stated preference-based methodology to estimate the marginal willingness to pay (MWTP) using a multidimensional approach [53]. Since this method can estimate the MWTP for each attribute, such as tourism facilities and services, it has been used in various tourism studies to evaluate the preference of tourists [49,50,51,52,53,54,55,56,57,58,59]. Therefore, CE was selected as the analysis method for understanding tourists’ preferences for child-friendly tourism facilities and services and developing a tourism destination management strategy for young children. 

For the experiments, the attributes and levels of child-friendly tourism facilities and services were employed based on previous studies as presented in Section 2 above. The specific attributes of young children’s tourism at Suwon Hwaseong Fortress consist of rest areas, restrooms and facilities designed for young children, children’s vehicles, and specialized courses, as shown in Table 1. An entrance fee was used as payment for the vehicles. Each attribute was divided into three levels. Level 1 for each attribute indicated the current state of the attribute or nothing. Levels 2 and 3 indicated alternative facilities and services which require more investment.

After collecting the choices of respondents, the indirect utility function was estimated by a conditional logit model (CL) designed by McFadden [53]. Using a maximum likelihood estimator for each attribute, MWTP was calculated. As all variables except the price variable (entrance fee) were binary variables, MWTP values for level 2 and level 3 of each attribute indicate additional WTP compared to level 1.

### 3.3. Questionnaire Design and Data Collection

The choice set for the experiment was composed of level-specific combinations of attributes from Table 1. Since there are too many choice profiles in the full factorial design (3^4^ × 4 = 324) to evaluate, a fractional factorial orthogonal design was used [60,61]. In this study, 16 profiles were used as the choice set for the experiment which were derived by an orthogonal design using SPSS 23.0. For each round, survey respondents could randomly select 3 of these 16 profiles, as shown in Table 2, and choose the most preferred alternative for them, or nothing. The experiment was conducted by all respondents over 5 rounds. The number of valid responses to the questionnaire was 374, and the total number of experiments was 1870.

The target population of this study was tourists in Suwon Hwaseong who were accompanied by young children under the age of 6. In order to increase the objectivity of the survey and to enhance the readability of the questionnaire items, a pretest with 35 responses was conducted in April 2019. After the questionnaire was revised, the main survey was conducted as an in-person 1:1 survey in Korean from April to May 2019 (peak season of outdoor recreation) by skilled interviewers. Sampling was performed using random sampling. A total of 500 questionnaires were distributed, and for a more accurate evaluation of facilities and services, the first visitor was excluded, and the final 374 samples were included in the analysis.

The questionnaire also included items regarding general characteristics of tourism with young children, tourism characteristics of young children in Suwon Hwaseong, and demographic characteristics of respondents. The time required for the survey was about 20 min.

## 4. Results

### 4.1. Demographic Characteristics of Respondents

The sample of 374 respondents with children included more women (n = 286, 76.5%) and mothers (n = 221, 59.1%). The average age of participants was 38.78 years (SD = 6.59) with more visitors in their 30 s (n = 237, 63.4%) than those in their 40 s (n = 100, 26.7%). College attendance/graduation was the most common educational level (n = 266, 71.1%) and household income was more than 4000 USD a month (n = 199, 53.2%), which indicated a high proportion of highly educated and high-income visitors.

### 4.2. Visitors’ MWTP for Child-Friendly Facilities and Services

The results of the CL model estimation of the responses of the selection experiments are shown in Table 3. First, the coefficient estimate of the price variable is negative and statistically significant at the 1% significance level. It indicates that rational decision-making—larger payment, lower choice probability—occurred in the experiment. The coefficient estimates for each attribute and level were significantly positive. It means that ceteris paribus, the probability of choosing alternatives (level 2 or 3) was higher against the current state (level 1).

Table 4 summarizes the MWTP calculation results based on the coefficient estimates of CL for each attribute and level. First, we confirmed the MWTP of an additional entrance fee of about 6000–9000 KRW for each attribute and level in general. Despite the relatively low MWTP for child-friendly restrooms and children’s vehicles, it is still more than 5000 KRW (=4.22 USD), which is still more than five times the current admission fee of 1000 KRW (=0.84 USD). Second, among the suggested attributes, the highest MWTP was seen for a specialized course that young children can actively enjoy (level 1 → 3). However, the difference in MWTP for each attribute was not statistically significant. Third, in general, the MWTP of the third level was higher than that of the second level, so the more investment in facilities and services is made, the higher the preference. Again, there was no statistically significant difference between levels. In the case of the rest areas, it was found that among the attributes belonging to the third level, indoor rest areas had less MWTP than in the second level.

## 5. Discussion

The present study seeks to establish a tourism destination management strategy centered on young children for accessible tourism from a public perspective. Preferences for facilities and services for young children were identified using CEs. The study site was Hwaseong Fortress, a UNESCO World Heritage Site, located in Suwon City, a leader in the clean restroom culture. The main implications of the study are as follows.

First, the WTP for child-friendly tourism facilities and services was high. According to existing research, the weak immune systems of young children leads to increased concerns about environmental hygiene and health. In addition, young children’s physical constraints lead to high demand for convenient mobility and comfortable rest [3,4]. The results show that the WTP for each attribute and level was about 5–9 USD. Considering that the admission fee for Hwaseong Fortress is about 1 USD, the high preference for facilities and services for young children was confirmed, as seen in previous studies [3,4,11,12]. A small fee is required for rides, but restrooms and rest areas are generally free. Nevertheless, the generally high WTP reflects the desire for improved facilities and services for young children.

Second, an examination of attributes indicated that the WTP was found in the decreasing order of specialized courses for young children, rest areas, restrooms, and vehicles. Respondents showed the highest WTP for level 3 of a specialized course for young children, which aims to develop a special zone or course that young children can actively enjoy. A key characteristic of young children’s tourism is to emphasize primary needs such as hygiene and safety [4]. In particular, it is necessary to engage young children who have difficulty understanding the historical value of Hwaseong Fortress in simple play activities and games that utilize amusing characters and colors, in order to stimulate their interests.

Third, WTP was higher for outdoor rest areas than indoor rest areas. The outdoor-oriented Hwaseong Fortress has rest areas in the form of unroofed benches that may not be appropriate as resting areas for young children since they do not shield out the sunlight and rain. Level 2 is an outdoor rest area that adds shades to existing outdoor benches, and level 3 is an indoor rest area equipped with air conditioning and air purifiers. The results indicate that level 2, the supplementation of existing outdoor rest areas, was most preferred. This preference implies that visitors to the outdoor tourist destination of Hwaseong Fortress prefer to relax in outdoor areas rather than stay indoors for long periods of time.

Fourth, with regards to restroom facilities, WTP was high for child-friendly restrooms including hot water, diaper changing stations, and child-friendly toilets and sinks. Although Suwon is a leader in restroom culture, these results point to insufficiencies in tourism facilities and services for young children. For example, more careful management of the height of sinks as mentioned in KTO’s guide is needed [36]. Interestingly, the preference for installation of young children’s facilities and services in both male and female restrooms was higher than for in female restrooms only. The recognition of only mothers as the main caregivers of young children, and the provision of child-friendly facilities and services in female restrooms only, imply the need for improvement in gender parity in young children’s care facilities.

Fifth, whereas MWTP for level 2 (convenience) and 3 (convenience and fun) for children’s vehicles was smaller than for other attributes, WTP was still five times that of the current admission fee. Nyman and others also cited accessibility as an important factor for travel decision-making for tourists with young children [40]. The difference between level 2 and level 3 was very small, and the preference for providing fun in addition to convenience was not large. The various linked resources within the open spaces of Hwaseong Fortress create long travel distances. As accompanying young children have limitations in walking long distances, as compared to adults, there is a large preference for rides that enhance convenience. The provision of ride services is particularly important at a destination such as Hwaseong Fortress, which has long travel distances and limited accessibility for young children. In addition, the hygienic management of these vehicles utilized by young children will be important, particularly in the era of COVID-19.

## 6. Conclusions

The present study has significant practical implications in that the preferences for facilities and services for young children at tourist destinations was estimated using CEs, and tourists’ priorities were identified from a public perspective. Previous studies on young children’s tourism focused mainly on the importance of tourism for young children, and key factors in tourist destination choices. In contrast, the present study examined more specifically preferences for levels of facilities and services as a key factor in choosing a tourist destination. This is useful in terms of tourism destination management for young children.

Due to the COVID-19 pandemic, the importance of health/hygiene management strategies is growing. Based on the results, the following suggestions for a tourism destination management strategy for young children can be provided. First, child-friendly restrooms have become a necessity, not just a preference, in a COVID-19 tourism environment in which cleanliness is most important [35]. In particular, convenience facilities for children should be installed in both men’s and women’s restrooms, not in women’s restrooms only. In addition, not only installation but also continuous cleanliness management is required.

Second, it is necessary to install rest areas suitable for outdoor tourist attractions. The existing resting space for children was mainly a private indoor space for baby feeding and diaper changing. However, in outdoor spaces, since the distance that children can move around is short and they have to take frequent breaks, the preference for basic rest areas with more benches and awnings was rather high. Therefore, maintenance of outdoor rest areas with improved convenience is required.

Third, the effort to minimize crowding during the COVID-19 era is emerging as a top priority at tourist destinations. Minimizing contact is particularly relevant for young children with weak immune systems. The present study showed the highest preference for specialized courses for young children. Not only can this lead to more attractive tourist destinations for young children, but also be a zoning strategy that separates adult tourists from young child tourists as a response to COVID-19. In addition, convenience vehicles such as strollers and wagons can also serve to provide a private space by separating young children from other adults.

The present study focused on child-friendly tourism facilities and services including restrooms, children’s vehicles, rest areas, and specialized courses. There are other important factors in the dimension of tourism destination management such as programs and information provision that need study. For example, preferences for various levels of information provision can be analyzed. It is possible to consider an information map or app for caregivers of young children. This can be used as a congestion guidance system to avoid congestion during the COVID-19 era. Other information provision methods that enable young children to easily understand and experience tourism resources, such as augmentative and alternative communication (AAC), are also available.

## Figures and Tables

**Table 1:** Attributes and levels for the Choice Experiment.

Attributes	Level	Description
Rest area	1	[Current] Outdoor bench
	2	Bench and awning (sunshade, rain shelter)
	3	Indoor rest area (air conditioning and heating, air purification, shelter from wind)
Restrooms and facilities designed for young children	1	[Current] Some facilities for young children are installed, mainly in women’s restrooms, but not enough ^1^
	2	Improvement of facilities designed for young children mainly in women’s restrooms
	3	Improvement of facilities designed for young children in both men’s and women’s restrooms
Children’s vehicles	1	[Current] None for young children
	2	Mobile vehicles (Stroller, Wagon)
	3	Mobile vehicles and play-type vehicles (hand push tricycles, push cars)
Specialized courses for young children and families	1	[Current] None for young children
	2	Guided course for young children (separate, safe, and low difficulty)
	3	Specialized course for young children to actively enjoy
Entrance fee (per person)	1	1000 KRW (0.84 USD)
	2	2000 KRW (1.69 USD)
	3	3000 KRW (2.53 USD)
	4	4000 KRW (3.38 USD)

^1^ Facilities designed for young children: Hot water, diaper changing stations, young children restrooms, (low) sinks for young children, child safety seats.

**Table 2:** Examples of cards with different levels of attributes in the choice experiment.

Attributes	Alternatives		
	Card 1	Card 2	Card 3
Rest area	(Level 1) Outdoor bench	(Level 3) Indoor rest area	(Level 1) Outdoor bench
Restrooms and facilities	(Level 1) Not improved	(Level 3) Improvement in both	(Level 2) Improvement in women’s only
Children’s vehicles	(Level 2) Mobile vehicles	(Level 1) None	(Level 3) Mobile vehicles and play-type vehicles
Specialized courses for young children	(Level 3) Specialized course	(Level 3) Specialized course	(Level 3) Specialized course
Entrance fee	2000 KRW	3000 KRW	4000 KRW

**Table 3:** Conditional logit model estimation results for child-friendly facilities and services.

Attributes and Levels		Coefficient ^1^
Entrance fee		−1.3 × 10^−4^ * (2.7 × 10^−5^)
Rest area	LV. 2	1.018 * (0.079)
	LV. 3	0.900 * (0.080)
Restrooms and facilities designed for young children	LV. 2	0.744 * (0.076)
	LV. 3	0.889 * (0.074)
Children’s vehicles	LV. 2	0.841 * (0.080)
	LV. 3	0.857 * (0.079)
Specialized courses for young children and families	LV. 2	0.926 * (0.081)
	LV. 3	1.207 * (0.080)
Log likelihood		−1642.9
Number of obs.		5457
LR χ^2^		710.9

^1^ Parentheses indicate standard errors, * *p* < 0.001. Table shows the conditional logit model estimation results of the selections.

**Table 4:** Marginal willingness to pay (MWTP) estimation results.

Attributes and Level		MWTP ^1^ [90% Confidence Interval] ^2^
Rest area	LV.1 → LV.2	7682 [5572–11,629]
	LV.1 → LV.3	6793 [4877–10,368]
Restrooms and facilities designed for young children	LV.1 → LV.2	5615 [3994–8659]
	LV.1 → LV.3	6708 [4880–10,190]
Children’s vehicles	LV.1 → LV.2	6348 [4586–9613]
	LV.1 → LV.3	6468 [4650–9820]
Specialized courses for young children and families	LV.1 → LV.2	6986 [5024–10,674]
	LV.1 → LV.3	9112 [6704–13,672]

^1^. Willingness to pay is a payment amount that one is willing to pay as content is added compared to level 1 (current status). ^2^. The 90% confidence interval was calculated using the Krinsky–Robb parametric bootstrap method, with 10,000 resamples [62].
